# Host transcriptome response to *Mycoplasma bovis* and bovine viral diarrhea virus in bovine tissues

**DOI:** 10.1186/s12864-025-11549-2

**Published:** 2025-04-10

**Authors:** Anna K. Goldkamp, Randy G. Atchison, Shollie M. Falkenberg, Rohana P. Dassanayake, John D. Neill, Eduardo Casas

**Affiliations:** 1 Ruminant Diseases and Immunology Research Unit, National Animal Disease Center, Agricultural Research Service, Department of Agriculture, Ames, IA USA; 2 Animal Plant Health Inspection Service, Department of Agriculture, Centers for Veterinary Biologics, Ames, IA USA; 3 College of Veterinary Medicine, Auburn University, Auburn, AL USA

**Keywords:** Bovine, Gene expression, Mycoplasma bovis, Bovine viral diarrhea virus

## Abstract

**Background:**

*Mycoplasma bovis* is a prominent pathogen associated with respiratory disease in livestock. Respiratory disease in cattle often involves co-infection, where a primary viral infection can weaken the host immune system and thus enhance subsequent bacterial infection. The objective of this study was to investigate changes in the host (cattle) transcriptome during bacterial-viral co-infection. RNA sequencing was done in whole blood cells (WBC), liver, mesenteric lymph node (MLN), tracheal-bronchial lymph node (TBLN), spleen, and thymus collected from Control animals (*n* = 2), animals infected with *M. bovis* (MB; *n* = 3), and animals infected with *M. bovis* and bovine viral diarrhea virus (BVDV) (Dual; *n* = 3).

**Results:**

Thymus and spleen had the greatest number of differentially expressed genes (DEGs) out of all tissues analyzed. In spleen, genes involved in maintenance of the extracellular matrix (ECM) including collagen type XV alpha 1 chain (*COL15A1*), collagen type IV alpha 2 chain (*COL4A2*), and heparan sulfate proteoglycan 2 (*HSPG2*) were the most significantly downregulated in Dual compared to Control and MB. In thymus, complement 3 (C3) was a highly significant DEG and upregulated in Dual compared to Control and MB. Interferon alpha inducible protein 6 (*IFI6*) and interferon-induced transmembrane proteins (*IFITM1* and *IFITM3*), were significantly associated with infection status and upregulated in spleen and thymus of Dual compared to Control and MB.

**Conclusion:**

Downregulation of ECM components may cause degradation of the ECM and contribute to increased viral spread due to co-infection. Hyperactivation of complement pathway genes may contribute to damage to the thymus and influence severity of co-infection. Co-expression of *IFI6*, *IFITM1* and *IFITM3* across lymphoid tissues may be connected to enhanced pathogenesis in co-infection. These findings suggest co-infection exacerbates disease severity through modulation of ECM components in spleen and complement and coagulation cascades in the thymus. These impacted pathways may underlie thymic atrophy and impaired pathogen clearance due to BVDV and *M. bovis* co-infection.

**Supplementary Information:**

The online version contains supplementary material available at 10.1186/s12864-025-11549-2.

## Introduction

Bovine respiratory disease (BRD) is a significant cause of mortality and morbidity in the beef industry, causing approximately 57% of mortality in United States feedlots [1]. Major clinical signs of BRD include nasal discharge, depression, fever, decreased appetite, and death [2]. Despite ongoing efforts to develop and implement treatments for BRD, annual economic losses are estimated to be more than one billion dollars in the United States [3, 4].

BRD is a multifactorial and multietiological disease complex. Management-related and environmental stressors can make an animal susceptible to a range of bacterial and viral pathogen infections [5]. The most common pathogens associated with BRD include: *Mannheimia haemolytica*, *Pasteurella multocida*, *M. bovis*, *Histophilis somni*, bovine herpes virus 1, bovine viral diarrhea virus 1 and 2 (BVDV-1 and − 2), bovine respiratory syncytial virus, and parainfluenza virus type 3. Because a variety of pathogens can cause BRD and there is a limited understanding in host immune response to infection, diagnosis and treatment of animals is challenging. Previous work has found synergistic interactions during co-infection of viral and bacterial pathogens [6, 7]. One study found that *M. bovis* was present in nearly 92% of animals with chronic antibiotic-resistant pneumonia and BVDV was detected in over half of the same cases [6]. Another study observed alterations in microRNA (miRNA) expression profiles due to co-infection with *M.* bovis and BVDV, suggesting miRNA-mediated changes in gene expression at the post-transcriptional level [7].

Previously, RNAseq was used to evaluate transcriptional responses in lung and four lymph node tissues (bronchial, retropharyngeal, nasopharyngeal, and pharyngeal tonsil) of animals challenged with pathogens associated with the BRD complex [8]. By comparing animals challenged with *M. bovis* or BVDV, the study showed that gene expression in lymphoid tissues differs significantly between viral and bacterial infections [8]. To date, there have been no studies investigating transcriptional regulation within host tissues during co-infection with *M. bovis* and BVDV. Therefore to fill this knowledge gap, we aimed to detect differential gene expression across tissues in Control, *M. bovis* (MB), and co-infected (Dual; *M. bovis* and BVDV) treatment groups. The objective of the study was to identify tissue-specific host immune responses due to single infection with MB and co-infection with MB and BVDV. Thus, RNA sequencing was done in thymus, spleen, tracheal-bronchial lymph node (TBLN), mesenteric lymph node (MLN), liver, and whole blood cells (WBC) across all three treatment groups.

## Materials and methods

### Animal welfare

Animals housed and samples collected for this study were handled in accordance with the Animal Welfare Act Amendments (7 U.S. Code e § 2131 to § 2156). All procedures were approved by the Institutional Animal Care and Use Committee of the National Animal Disease Center (ARS-2016-581). Intravenous injection of sodium pentobarbital was used to humanely euthanize animals following per label dose and the discretion of the clinical veterinarian.

### Animal study

Holstein male calves (~ 2 months of age) were assigned to one of three treatment groups: Control (*n* = 2), *M. bovis* (MB; *n* = 3), and Dual infection with MB and BVDV (Dual; *n* = 3). Animals were purchased from a private vendor in Iowa. On day 0, MB calves were inoculated with *M. bovis* and Dual calves with BVDV. Control calves were given 5 mL of cell culture supernatant of uninfected cells. On day 6, Dual calves were inoculated with *M. bovis*. *M. bovis* inoculation was done 6 days after BVDV inoculation in Dual calves to maximize the susceptibility of the calf to a secondary *M. bovis* infection. Inoculums were administered intranasally to calves using a mucosal atomization device (Teleflex, Morisville, NC) attached to a 10 mL syringe.

The BVDV isolate (RS886) used for the study was a noncytopathic BVDV type 2 strain and was isolated and propagated at the National Animal Disease Center, as previously described [9–12]. The *M. bovis* isolate (KRB5) used in this study was originally cultured in 2016 from the lung of a calf with pneumonia. KRB5 was grown and prepared, as previously described [13, 14]. Each calf received 5 mL of *M. bovis* inoculum containing a total of 1 × 10^11^ colony forming units. For BVDV inoculation, each calf received 5 mL of BVDV inoculum containing a total of 5 × 10^6^ TCID50.

17 days after initial infection and 11 days after Dual *M. bovis* infection, calves were euthanized. Whole blood cells (WBC), liver, mesenteric lymph node (MLN), tracheal-bronchial lymph node (TBLN), spleen, and thymus, were collected at necropsy. WBC was collected by venipuncture into PAXgene tubes (PreAnalyliX GmbH, Hombrechtichon, Zurich, Switzerland). All other tissue samples were perfused with RNAlater-ICE (Thermo Fisher Scientific, Waltham, MA, USA), snap frozen in an ethanol dry ice bath and stored at -80˚C until RNA extraction.

### RNA isolation

Total RNA was extracted from all samples with the mirVana total RNA isolation kit (Thermo Fisher Scientific, Waltham, MA). Concentration and RNA integrity number were evaluated using an Agilent 2100 Bioanalyzer Eukaryote Total RNA Nanochip (Agilent Technologies, Santa Clara, CA, United States). RNA integrity numbers were ≥ 7.5 for all samples.

### Library preparation and sequencing

Libraries were prepared using the NEBNext Ultra mRNA library prep kit mRNA magnetic isolation module according to manufacturer’s instructions (New England Biolabs, Ipswich, MA, United States). Multiplexing was done with the NEBNext Multiplex Oligos for Illumina kit. Library concentration and quality was assessed (library sizes of ~ 300 bp) with the Agilent 2100 Bioanalyzer High Sensitivity DNA chip (Agilent Technologies). Libraries were pooled in equal concentration and further concentrated using the QiaQuick PCR clean up kit (Qiagen, Germantown, MD, United States). The resulting pooled library was stored at -20 °C until sequencing on the Illumina HiSeq 3000 System (2 × 100 bp) (Illumina, San Diego, CA, United States).

### Processing and mapping of RNAseq data

To evaluate raw sequences before and after trimming, FastQC (v 0.12.1) was used. Adapter sequences were trimmed from raw reads and low-quality sequences were removed (quality < 30, minimum length of 60) using Cutadapt (v 4.0) [15]. Trimmed reads were aligned to the bovine reference genome, ARS UCD1.2, using Hisat2 (v 2.2.1) with the following adjusted parameters: --score-min L,0,-0,2 [16. The Hisat2 python script (extract_splice_sites.py) was also used to extract known splice sites and the resulting file used as input (known-splicesite-infile) to increase sensitivity in mapping spliced reads. The featureCounts function of subread (v 2.0.4) was used to generate count tables with adjusted parameters (--countReadPairs -M) [17].

### Differential expression and enrichment analysis

Differential expression analysis was done with DESeq2 and a DESeq dataset object was created using the DESeqDataSetFromMatrix function [18]. The median of ratios method of DESeq2 was used for data normalization and differential expression was determined using a negative binomial GLM and Wald test statistics. Genes with an adjusted *p*-value ≤ 0.05 were classified as differentially expressed.

Heatmaps were generated with the pheatmap package of R and logCPM transformation of RNAseq raw count data was done using the counts per million (CPM) method from edgeR for visualization. Functional enrichment analysis of differentially expressed genes, such as molecular function, biological processes, and KEGG pathways was performed using Database for Annotation, Visualization, and Integrated Discovery (DAVID) (https://david.ncifcrf.gov/) [19]. Gene ontology terms with a *p*-value ≤ 0.05 were deemed significant. Principal component analysis (PCA), volcano, and enrichment plots were created with ggplot in R. Upset plots were created using the UpSetR package in R. Interferon-stimulated genes and regulation by interferon types was retrieved from the Interferome database (version 2.01) and based on high-throughput expression datasets in mouse and human (https://interferome.org) [20].

### Weighted gene co-expression analysis (WGCNA)

To evaluate the correlation between gene expression and infection status, the WGCNA R package (version 1.72-5) was used to identify co-expressed genes within each treatment group. The top 25% of genes with high expression variance were retained for further analysis using the quantile function of R. The correlation between the expression of all pairs of genes was used to create a correlation adjacency matrix with the adjacency function of WGCNA. To filter for strongly connected genes, the correlation values were transformed with a soft threshold power of 25 that equates to a scale-free topology index of (R [2]) 0.9. Hierarchical clustering with the hclust function was then used to group genes into modules that show similar expression patterns with a minimum module size of 30 genes. Eigen values for each module were calculated using the moduleEigengenes function of WGCNA, where the module eigen value acts as a representative of gene expression in the module. The resulting eigen values were correlated to infection status and correlation *p*-values were calculated using the corPvalueStudent function of WGCNA. For Module III, the correlation between gene expression and infection status, known as gene significance, was calculated. In addition, the correlation between gene expression and the eigen value of each model was termed module membership.

## Results

### Transcriptome sequencing

To evaluate transcriptomic responses from calves used in each respective treatment group, RNA sequencing was done in samples collected from liver, spleen, thymus, MLN, TBLN, and WBC. On average, there were 30,598,153 raw reads and 29,565,873 clean reads (adapter and quality trimmed reads) per sample. Clean reads were mapped to the bovine reference genome with an average alignment rate of 97.2% (liver), 95% (spleen), 95.5% (thymus), 95.5% (MLN), 94.8% (TBLN), and 85% (WBC) (Supplementary Table S1 & S2).

**Fig. 1:**
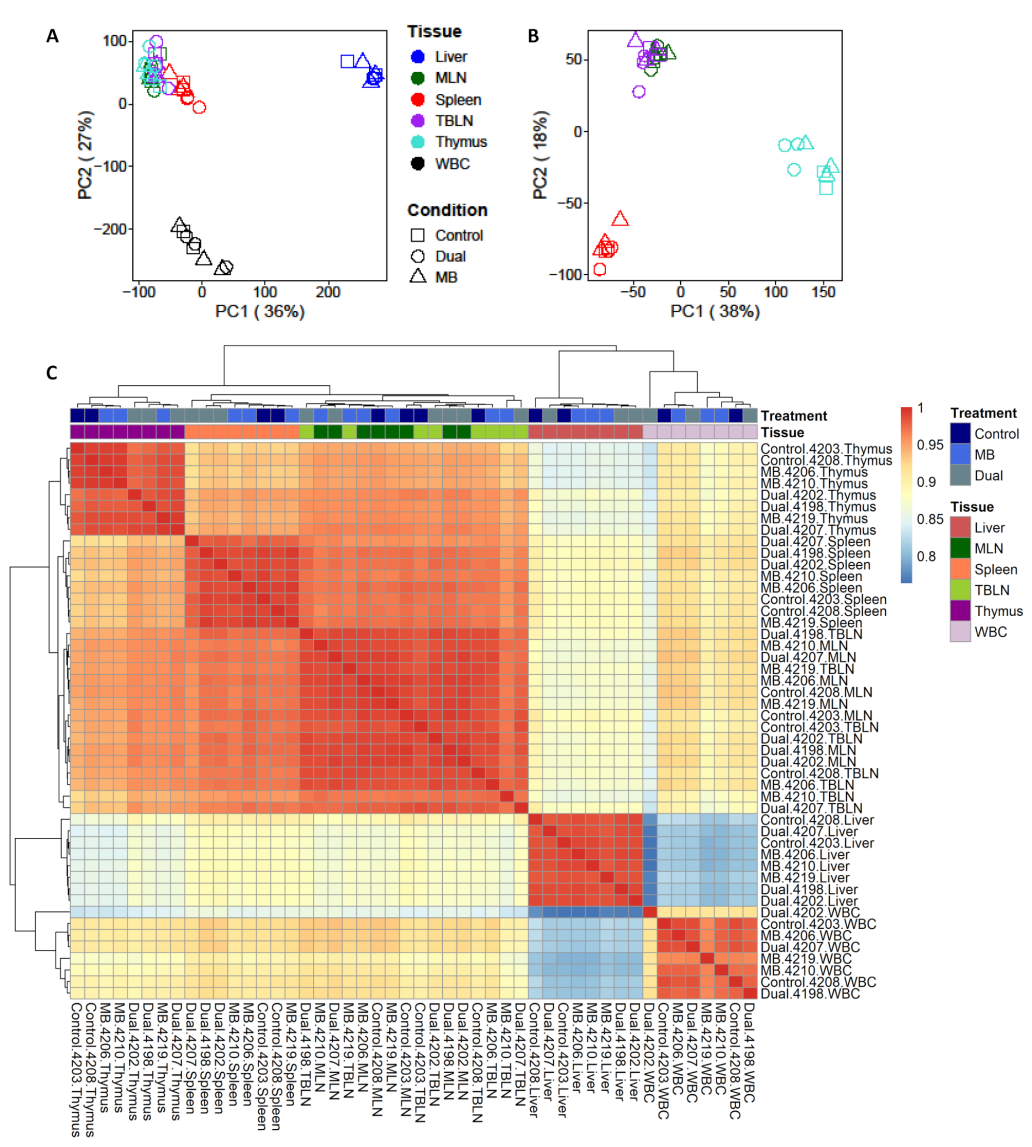
Principal component analysis (PCA) and correlation heatmap plots across all samples and tissues. (**A**) PCA plots for all samples analyzed. (**B**) PCA plots for all samples, excluding liver and WBC. (**C**) Correlation heatmap for all samples. Red and blue colors represent the highest and lowest correlation values, respectively. Tissues are highlighted in different colors and samples labeled on x- and y-axis. Samples belonging to Control, *M. bovis* (MB), and Dual groups as well as those derived from liver, mesenteric lymph node (MLN), serum, spleen, tracheal-bronchial lymph node (TBLN), thymus, and whole blood cells (WBC) are shown in different colors

Principal component analysis (PCA) of all samples showed clustering by tissue instead of experimental treatment group (Fig. 1A). Principal component 1 (PC1) and 2 (PC2) captured 36% and 27% of gene expression variance, respectively. Liver and WBC formed their own clusters, separate from lymphoid tissues (MLN, TBLN, spleen, and thymus). An additional PCA, excluding liver and WBC, indicated that lymph node samples (MLN and TBLN) had greater similarity in gene expression compared to thymus and spleen (Fig. 1B). These findings were also supported by correlation analysis (Fig. 1C). Treatment-specific differences in gene expression were evaluated for each tissue, which showed that the Dual group in thymus and spleen had distinct expression compared to Control and MB groups.

### Differentially expressed genes in response to *M. bovis* and co-infection

**Fig. 2:**
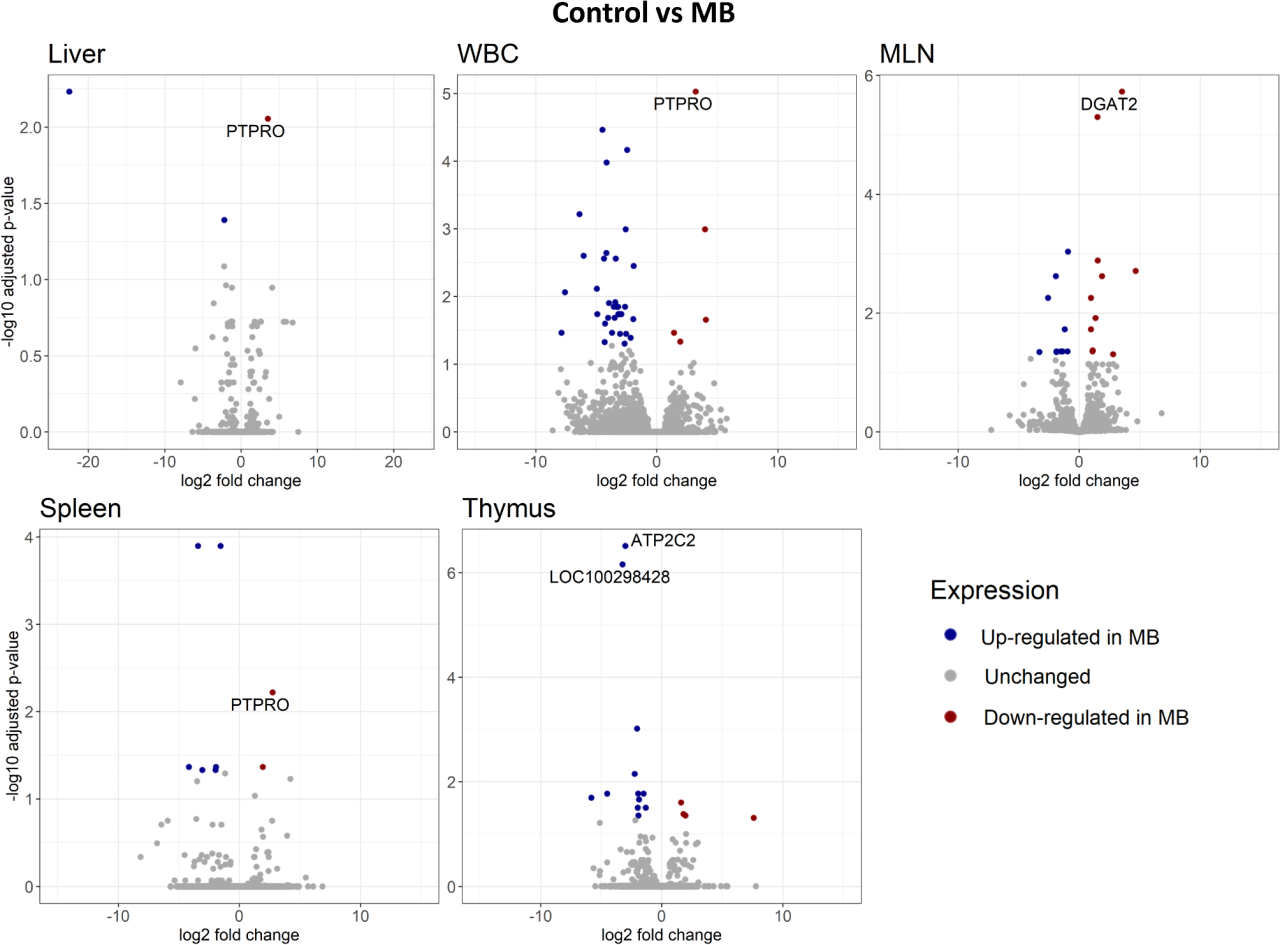
Volcano plots of differentially expressed genes (DEGs) between Control and *M. bovis* (MB) in liver, whole blood cell (WBC), mesenteric lymph node (MLN), spleen, and thymus. The x-axis indicates log2 foldchange and the y-axis indicates -log10 adjusted *p*-value for each DEG. DEGs with an adjusted *p*-value < 0.05 were deemed significant. Upregulated DEGs are shown in blue. Downregulated DEGs are shown in red. Non-significant genes are shown in grey. PTPRO = protein tyrosine phosphatase receptor type O; DGAT2 = diacylglycerol O-acyltransferase 2; ATP2C2 = ATPase secretory pathway Ca2 + transporting 2; LOC100298428 = uncharacterized LOC100298428

The number of differentially expressed genes (DEGs) for each comparison varied across tissue type with the fewest DEGs in Control vs. MB comparisons (Table 1). Protein tyrosine phosphatase receptor type O (*PTPRO*) was a significant DEG in liver, WBC, and spleen in Control vs. MB (Fig. 2). Downregulation of *PTPRO* in MB compared to Control groups was observed in these tissues.

**Table 1:** Number of up- and down-regulated differentially expressed genes (DEGs) for each treatment group in contrast to control (Control vs. *M. bovis* (MB); control vs. Dual) or MB (MB vs. Dual). Number of DEGs are shown for whole blood cell (WBC), liver, mesenteric lymph node (MLN), tracheal-bronchial lymph node (TBLN), spleen, and thymus

		No. of DEGs			
Tissue	Comparison	Up-regulated	Down-regulated	Total	Regulation Reference*
WBC	Control vs. MB	5	33	38	Control
	Control vs. Dual	5	5	10	Control
	MB vs. Dual	17	7	24	MB
Liver	Control vs. MB	1	2	3	Control
	Control vs. Dual	23	27	50	Control
	MB vs. Dual	17	22	39	MB
MLN	Control vs. MB	11	10	21	Control
	Control vs. Dual	17	2	19	Control
	MB vs. Dual	32	7	39	MB
TBLN	Control vs. MB	**-**	**-**	**-**	Control
	Control vs. Dual	6	27	33	Control
	MB vs. Dual	6	2	8	MB
Spleen	Control vs. MB	2	6	8	Control
	Control vs. Dual	127	25	152	Control
	MB vs. Dual	322	162	484	MB
Thymus	Control vs. MB	4	12	16	Control
	Control vs. Dual	1350	1898	3248	Control
	MB vs. Dual	524	949	1473	MB

*Regulation reference refers to the group that the DEGs are up- or down-regulated in. For Control vs. MB and Control vs. Dual, up-regulated and down-regulated columns indicate number of differentially expressed genes in Control compared to MB or Dual. For MB vs. Dual, up-regulated and down-regulated columns indicate number of differentially expressed genes in MB compared to Dual

The greatest number of DEGs were found in comparisons with the Dual group, as shown in Table 1. The number of DEGs in Control vs. Dual analyses ranged from 10 total DEGs in WBC to 3,248 total DEGs in thymus. In MB vs. Dual analyses, the total number of DEGs ranged from 8 total DEGs in TBLN to 1,473 DEGs in thymus. The full DESeq2 output for all comparisons in each tissue is shown in Supplementary Table S3.

**Fig. 3:**
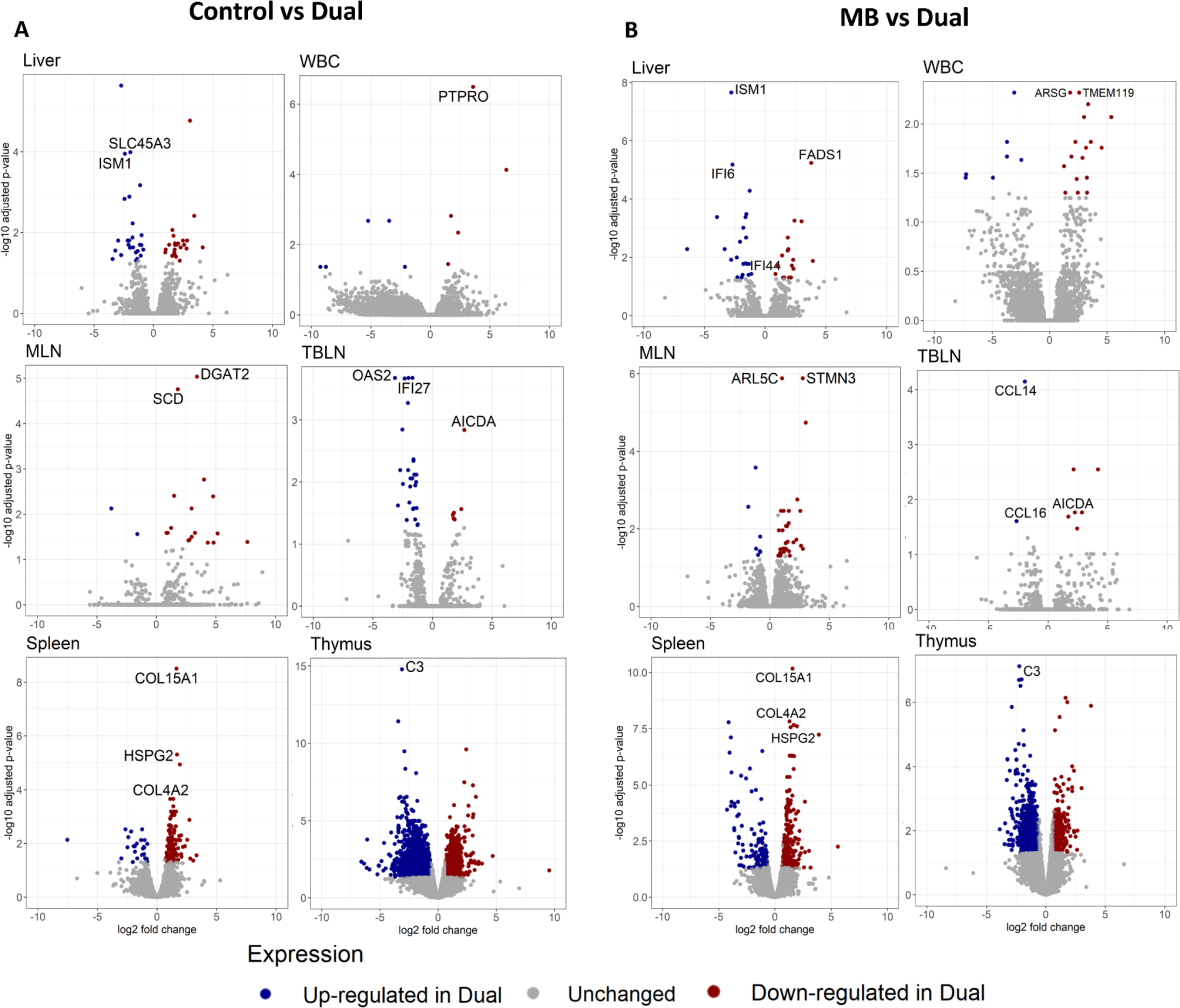
Volcano plots of differentially expressed genes (DEGs) between (**A**) Control vs Dual and (**B**) *M. bovis* (MB) vs Dual in liver, whole blood cell (WBC), mesenteric lymph node (MLN), tracheal-bronchial lymph node (TBLN), spleen, and thymus. The x-axis indicates log2 foldchange and the y-axis indicates -log10 adjusted *p*-value for each DEG. DEGs with an adjusted *p*-value < 0.05 were deemed significant. Upregulated DEGs are shown in blue. Downregulated DEGs are shown in red. Non-significant genes are shown in grey. SLC45A3 = solute carrier family 45 member 3; ISM1 = isthmin 1; PTPRO = protein tyrosine phosphatase receptor type O; IFI6 = interferon alpha inducible protein 6; FADS1 = fatty acid desaturase 1; ARSG = arylsulfatase G; TMEM119 = transmembrane protein 119; SCD = stearoyl-CoA desaturase; DGAT2 = diacylglycerol O-acyltransferase 2; OAS2 = 2’-5’-oligoadenylate synthetase 2; IFI27 = interferon alpha inducible protein 27; AICDA = activation induced cytidine deaminase; ARL5C = ARF like GTPase 5 C; STMN3 = stathmin 3; CCL14/16 = chemokine ligand 14/16; COL15A1 = collagen type XV alpha 1 chain; HSPG2 = heparan sulfate proteoglycan 2; COL4A2 = collagen type IV alpha 2 chain; C3 = complement C3

The DEGs for comparisons with the Dual group can be seen in Fig. 3. Among these genes, several were interferon-stimulated genes and regulated by Type I and II Interferons [20]. For example, 2`,5` oligoadenylate synthetase 2 (*OAS2*) in TBLN and solute carrier family 45 member 3 (*SLC45A3*) in liver were interferon-regulated and upregulated in Dual compared to Control (Fig. 3A). TBLN and liver showed downregulation of interferon-stimulated genes in the Dual group compared to MB, where activation induced cytidine deaminase (*AICDA*) was downregulated in TBLN and fatty acid desaturase 1 (*FADS1*) was downregulated in liver (Fig. 3B). In addition, interferon alpha inducible protein 6 (*IFI6)* and interferon induced protein 44 (*IFI44*) were upregulated in the Dual group in liver and chemokine ligands 14 and 16 (*CCL14* and *CCL16*) in TBLN compared to MB (Fig. 3B). Upregulation of *IFI6*, *IFI44*, and interferon alpha inducible protein 27 (*IFI27*) was also found in Dual compared to Control in TBLN. Upregulation of an adipokine, isthmin (*ISM1*), was found in liver of the Dual group compared to MB and Control (Fig. 3A and B).

Thymus and spleen had the greatest number of DEGs among all tissues due to single or Dual infection (Fig. 3A and B, **bottom**). In spleen, genes such as collagen type XV alpha 1 chain (*COL15A1*), collagen type IV alpha 2 chain (*COL4A2*), and heparan sulfate proteoglycan 2 (*HSPG2*), which play a role in maintaining the basement membrane of the extracellular matrix (ECM) for defense against infection, were among the downregulated genes with the most significance in Dual compared to Control and MB. An activator of the complement system, complement 3 (*C3*), was a highly significant DEG in thymus and was upregulated in Dual compared to Control and MB.

**Fig. 4:**
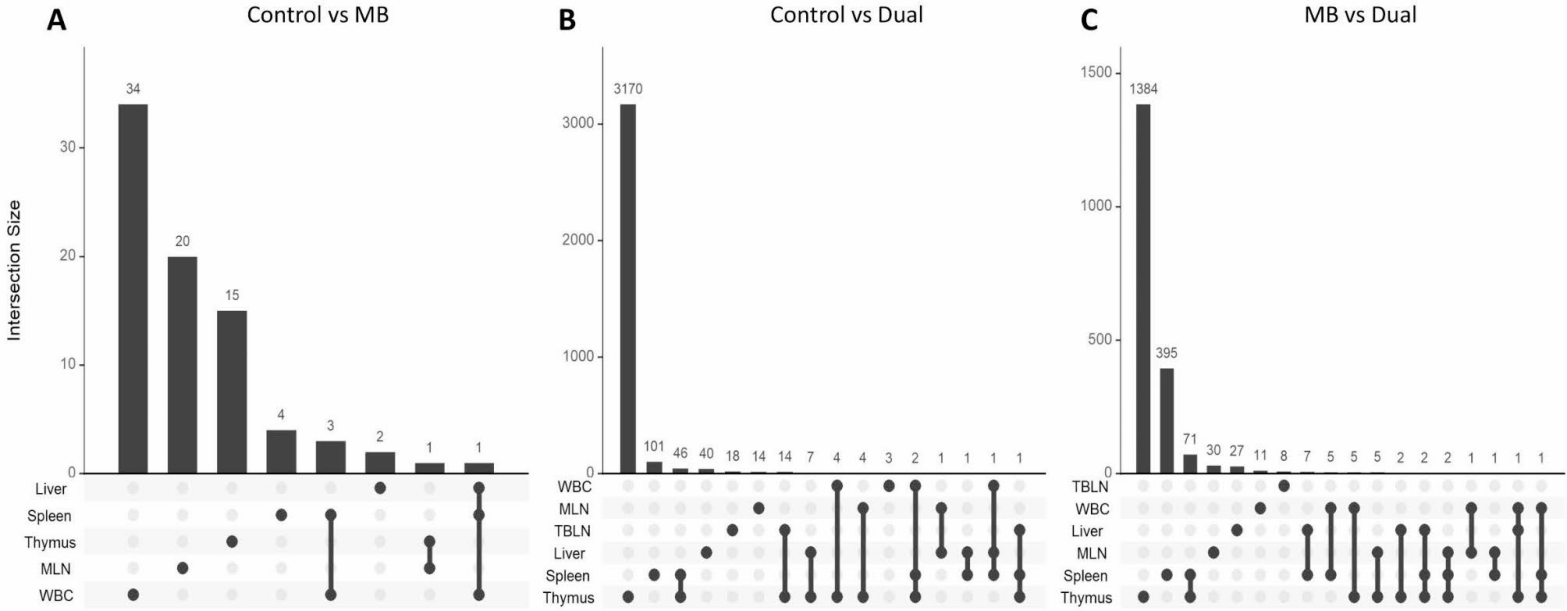
Intersection plots of unique and shared differentially expressed genes (DEGs) across tissues. Intersection plots in (**A**) Control vs. *M. bovis* (MB), (**B**) Control vs. Dual, and (**C**) MB vs. Dual for DEGs found in liver, spleen, thymus, mesenteric lymph node (MLN), tracheal-bronchial lymph node (TBLN), and whole blood cell (WBC). Individual dots under the bar graph indicate DEGs unique to a tissue. More than one dot under a bar indicates that the DEG is shared by the dotted tissues

Common differential expression patterns between tissues were also assessed. Thymus and spleen had 46 shared DEGs in Control vs. Dual and 71 in MB vs. Dual, which was the greatest among all tissue comparisons (Fig. 4B and C). Aside from thymus and spleen, few genes were differentially expressed in more than one tissue in Control vs. MB (Fig. 4A), Control vs. Dual (Fig. 4B), and MB vs. Dual (Fig. 4C). There were 1,384 and 395 DEGs that were unique to thymus and spleen in MB vs. Dual, respectively.

### Pathway enrichment analysis of differentially expressed genes in MLN, TBLN, WBC, and liver

As shown in Table 2, functional annotation showed significant pathway enrichment of DEGs for MLN, TBLN, WBC, and liver. Downregulation of genes involved in fatty acid synthesis was observed in MLN (stearoyl-coA desaturase/*SCD*, fatty acid synthase/*FASN*, and fatty acid elongase 6/*ELOVL6*) and liver (*FASN* and fatty acid desaturase 1/*FADS1*) of the Dual group compared to Control. *FASN* and *FADS1* were also downregulated in liver of the Dual group compared to MB. In addition, upregulation of genes involved in amino acid metabolism was found in the liver of the Dual group compared to Control. For Control vs. Dual, dysregulated genes in TBLN were associated with pathways involved in infection by viruses (Influenza A and Coronavirus disease) and parasites (African Trypanosomiasis).

**Table 2:** Pathways significantly enriched with differentially expressed genes (DEGs) in mesenteric lymph node (MLN), tracheal bronchial lymph node (TBLN), whole blood cell (WBC), and liver. Enriched pathways with a *p*-value < 0.05 are shown for each tissue and each pairwise comparison (Control vs. *M. bovis* (MB); control vs. Dual; MB vs. Dual). Rows marked with– indicate that there was non-significant enrichment in the tissue comparison

Comparison	Tissue	Pathway	Count	*P*-Value	DEGs
Control vs. MB	WBC	**-**	-	-	-
Control vs. MB	Liver	-	-	-	-
Control vs. MB	TBLN	-	-	-	-
Control vs. MB	MLN	bta00561:Glycerolipid metabolism	3	1.67E-03	*DGAT2*,* LPL*,* PNPLA2*
MB vs. Dual	WBC	bta05330:Allograft rejection	2	2.64E-02	*LOC508646*,* BOLA-DOB*
		bta04940:Type I diabetes mellitus	2	2.74E-02	*LOC508646*,* BOLA-DOB*
		bta05332:Graft-versus-host disease	2	2.99E-02	*LOC508646*,* BOLA-DOB*
		bta05320:Autoimmune thyroid disease	2	3.49E-02	*LOC508646*,* BOLA-DOB*
MB vs. Dual	Liver	bta01212:Fatty acid metabolism	3	5.17E-03	*FASN*,* ACACA*,* FADS1*
		bta03320:PPAR signaling pathway	3	1.02E-02	*PLIN4*,* ANGPTL4*,* RXRG*
		bta00061:Fatty acid biosynthesis	2	3.27E-02	*FASN*,* ACACA*
		bta05160:Hepatitis C	3	4.35E-02	*OAS1X*,* OAS1Y*,* CLDN15*
MB vs. Dual	TBLN	bta04061:Viral protein interaction with cytokine and cytokine receptor	2	3.80E-02	*CCL14*,* CCL16*
MB vs. Dual	MLN	-	-	-	*-*
Control vs. Dual	WBC	-	-	-	*-*
Control vs. Dual	Liver	bta03320:PPAR signaling pathway	4	9.63E-04	*PLIN4*,* ANGPTL4*,* CPT1B*,* RXRG*
		bta00260:Glycine, serine and threonine metabolism	3	5.61E-03	*BHMT*,* LOC112443696*,* GNMT*
		bta00270:Cysteine and methionine metabolism	3	7.06E-03	*BHMT*,* LOC112443696*,* GNMT*
		bta01212:Fatty acid metabolism	3	8.38E-03	*FASN*,* CPT1B*,* FADS1*
		bta01100:Metabolic pathways	9	2.89E-02	*MIOX*,* BHMT*,* LOC112443696*,* FASN*,* GDPD1*,* AK4*,* INMT*,* FADS1*,* GNMT*
		bta04152:AMPK signaling pathway	3	3.51E-02	*SREBF1*,* FASN*,* CPT1B*
Control vs. Dual	TBLN	bta05143:African trypanosomiasis	3	2.78E-03	*HBA*,* HBA1*,* IDO1*
		bta05171:Coronavirus disease - COVID-19	4	1.93E-02	*OAS2*,* MX1*,* ISG15*,* C2*
		bta05164:Influenza A	3	4.97E-02	*OAS2*,* MX1*,* TNFSF10*
Control vs. Dual	MLN	bta01212:Fatty acid metabolism	3	2.28E-03	*SCD*,* FASN*,* ELOVL6*
		bta00561:Glycerolipid metabolism	3	3.02E-03	*DGAT2*,* GPAM*,* LPL*
		bta04152:AMPK signaling pathway	3	1.00E-02	*SCD*,* LEP*,* FASN*
		bta00360:Phenylalanine metabolism	2	2.43E-02	*GAT*,* LOC112441481*
		bta01040:Biosynthesis of unsaturated fatty acids	2	3.63E-02	*SCD*,* ELOVL6*

In WBC, *LOC508646* (ortholog of granzyme B) was upregulated and bovine leukocyte antigen, class II, DO beta (*BOLA-DOB*) was downregulated in Dual compared to MB, where both were enriched in pathways associated with autoimmune diseases (Table 2). For TBLN, chemokine ligands (*CCL14* and *CCL16*) were enriched in viral protein interaction with cytokines and chemokine signaling pathways, where both were upregulated in Dual compared to MB. Liver DEGs associated with Hepatitis C, including, claudin 15 (*CLDN15*) and 2`, 5` oligoadenylate synthetase 1 genes (*OAS1X* and *OAS1Y*), were upregulated in Dual compared to MB.

### Gene regulation of pathways enriched in thymus and spleen

**Fig. 5:**
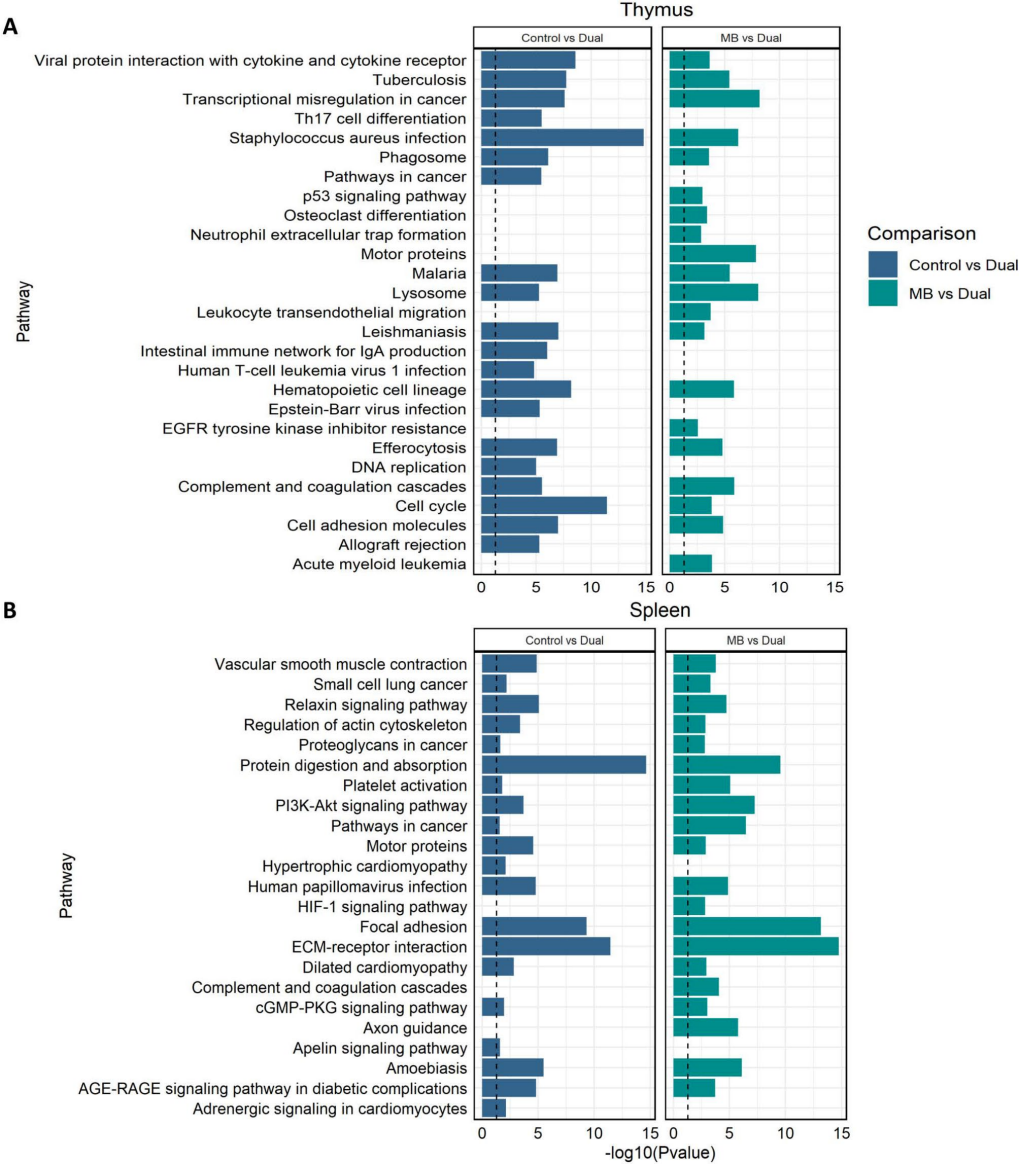
Pathways significantly enriched for differentially expressed genes. A subset of impacted biological processes associated with DEGs in Control vs. Dual and *M. bovis* (MB) vs. Dual comparisons in **(A)** Thymus and **(B)** Spleen. Gene ontology (GO) enrichment analysis was performed with DAVID and pathways with a *p*-value < 0.05 (dashed line) were considered significant. Pathways are shown in the y-axis and the -log10 adjusted *p*-value on the x-axis

The greatest number of significantly (*p*-value ≤ 0.05) enriched pathways was found in comparisons for spleen and thymus. For thymus, the top five most significant pathways for DEGs between Control vs. Dual were *Staphylococcus aureus* infection, Cell cycle, viral protein interaction with cytokine and cytokine receptor, hematopoietic cell lineage, and tuberculosis (Fig. 5A, **left**). For spleen, the top five most significant pathways in Control vs. Dual included: Protein digestion and absorption, ECM-receptor interaction, focal adhesion, amoebiasis, and Relaxin signaling pathway (Fig. 5B, **left**).

For DEGs found between MB and Dual, the top five most significant pathways in thymus were transcriptional misregulation in cancer, lysosome, motor proteins, *S. aureus* infection, and complement and coagulation cascades (Fig. 5A, **right**). In spleen, the top five most significant pathways for DEGs in MB vs. Dual included: ECM-receptor interaction, focal adhesion, protein digestion and absorption, PI3K/AKT signaling pathway, and pathways in cancer (Fig. 5B, **right**).

**Fig. 6:**
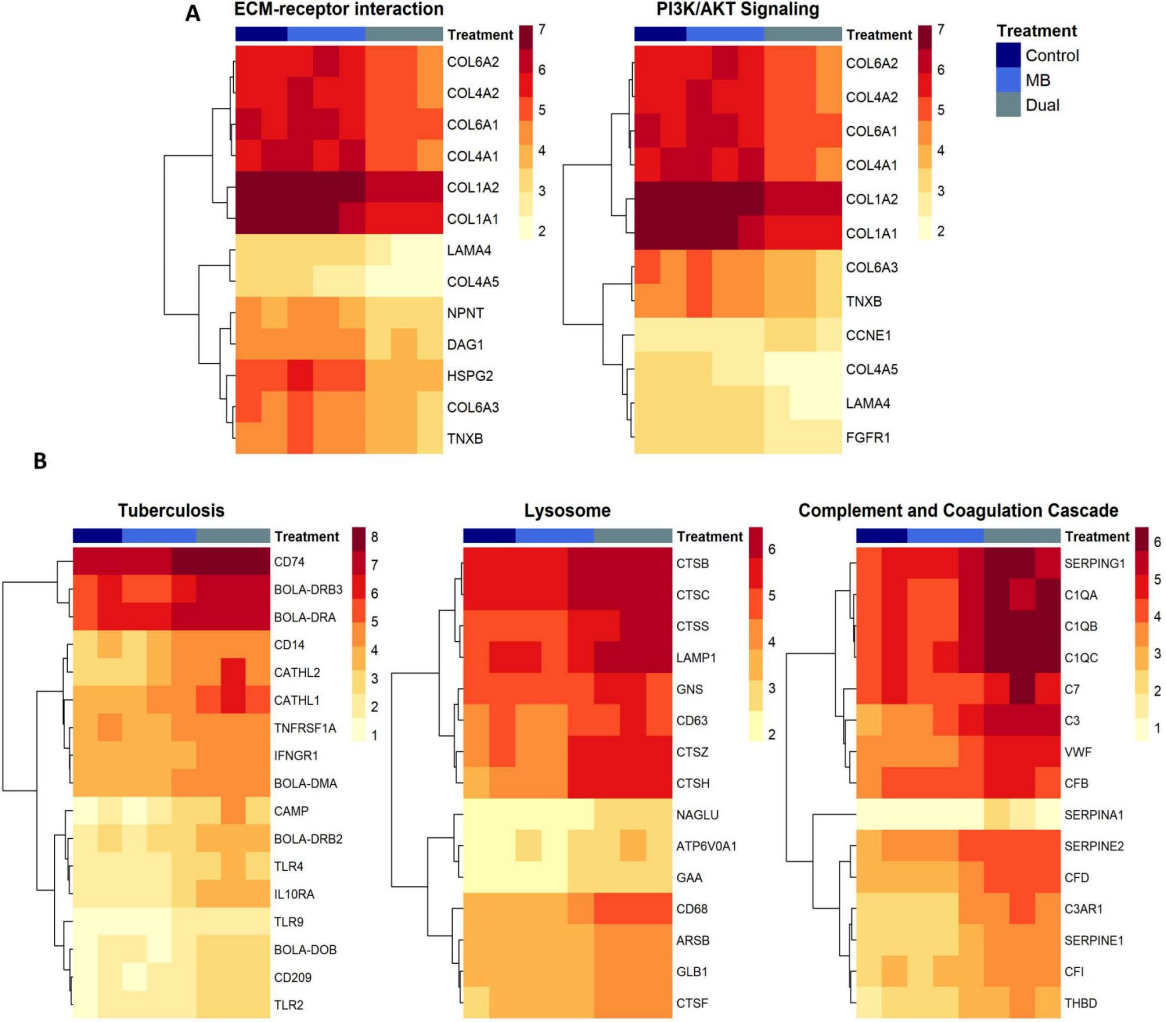
Heatmaps of shared differentially expressed genes (DEGs) in Control vs. Dual and *M. bovis* (MB) vs. Dual comparisons within impacted pathways in (**A**) spleen and (**B**) thymus. The color scale indicates the magnitude of expression (log counts per million (logCPM)) of the respective gene across samples

The ECM maintains tissue structure and function, where interactions between cell surface receptors and the ECM play a role in cell adhesion, migration, proliferation, and apoptosis [21]. Transcriptome analysis in spleen showed that genes involved in ECM-receptor interaction were downregulated in Dual compared to Control and MB. (Fig. 6A). Some of these downregulated genes (Type I, IV, and VI collagens, laminin 4/*LAMA4*, and tenascin XB/*TNXB*), are also involved in the phosphatidylinositol 3-kinase (PI3K)/ protein kinase B (AKT) signaling pathway. In addition, decreased expression of a fibroblast growth factor receptor (*FGFR1*) is observed in the Dual group in spleen, where expression of FGF receptors is necessary for AKT activation (Fig. 6A).

Differential expression analysis in thymus showed that genes involved in tuberculosis were upregulated in the Dual group compared to Control and MB (Fig. 6B, **left**). Clusters of differentiation (*CD14*, *CD74*, and *CD209*) and major histocompatibility complex class II genes (*BOLA-DRB2*, *BOLA-DRB3*, *BOLA-DRA*, *BOLA-DOB*, and *BOLA-DMA*) were upregulated in thymus of the Dual group compared to Control and MB (Fig. 6B). In addition, toll-like receptors are suggested to induce expression of antimicrobial peptides called cathelicidins during tuberculosis infection, and the present work showed increased expression of toll-like receptors (*TLR2*,* TLR4*, and *TLR9*) and cathelicidin genes (*CAMP*, *CATHL1*, *CATHL2*) in Dual compared to Control and MB [22]. The receptors for genes previously associated with thymic atrophy were upregulated in the Dual group, including interleukin-10 receptor subunit alpha (*IL10RA*), interferon-gamma receptor 1 (*IFNGR1*), and tumor necrosis factor receptor superfamily member 1 A (*TNFRSF1A*), compared to Control and MB.

Lysosomes function as the cell’s digestive compartment and support key events in immune response such as antigen processing and TLR activation. Increased expression of lysosome-associated genes was found in thymus of the Dual group, where there was an upregulation of cathepsins (*CTSB*, *CTSC*, *CTSF*, *CTSS*,* CTSH*, and *CTSZ*), glycosidases (*GLB1*, *GAA*, and *NAGLU*), sulfatases (*GNS* and *ARSB*) and lysosome markers (*LAMP1*, *CD63* and *CD68*) compared to Control and MB (Fig. 6B, **middle**). A vacuolar-ATPase (*ATP6V0A1*), which is a suggested co-factor of SARS-CoV-2 infection, was also an upregulated gene in the Dual group [23].

The complement and coagulation cascade pathway acts as a mediator to host defense against pathogens, where its activation leads to the production of molecules central to immunity and clotting. The genes that encode complement component 1q (*C1QA*, *C1QB*, and *C1QC*), which is the first protein in the complement cascade that directly binds to the surface of a pathogen, were upregulated in the Dual group (Fig. 6B, **right**) [24, 25]. Upregulation of complement components (*C3* and *C7*), a complement component receptor (*C3AR1*) and complement factors (*CFB*, *CFD*, and *CFI*) of the complement cascade was also found in the thymus of Dual individuals [26]. Markers of endothelial cell damage, Von Willebrand factor (*VWF*) and thrombomodulin (*THBD*), were upregulated in the Dual group. Several serpin genes (*SERPINA1*, *SERPINE1*, *SERPINE2*, and *SERPING1*) were also upregulated in Dual compared to Control and MB.

### Co-expressed genes associated with infection status in lymphoid tissues

**Fig. 7:**
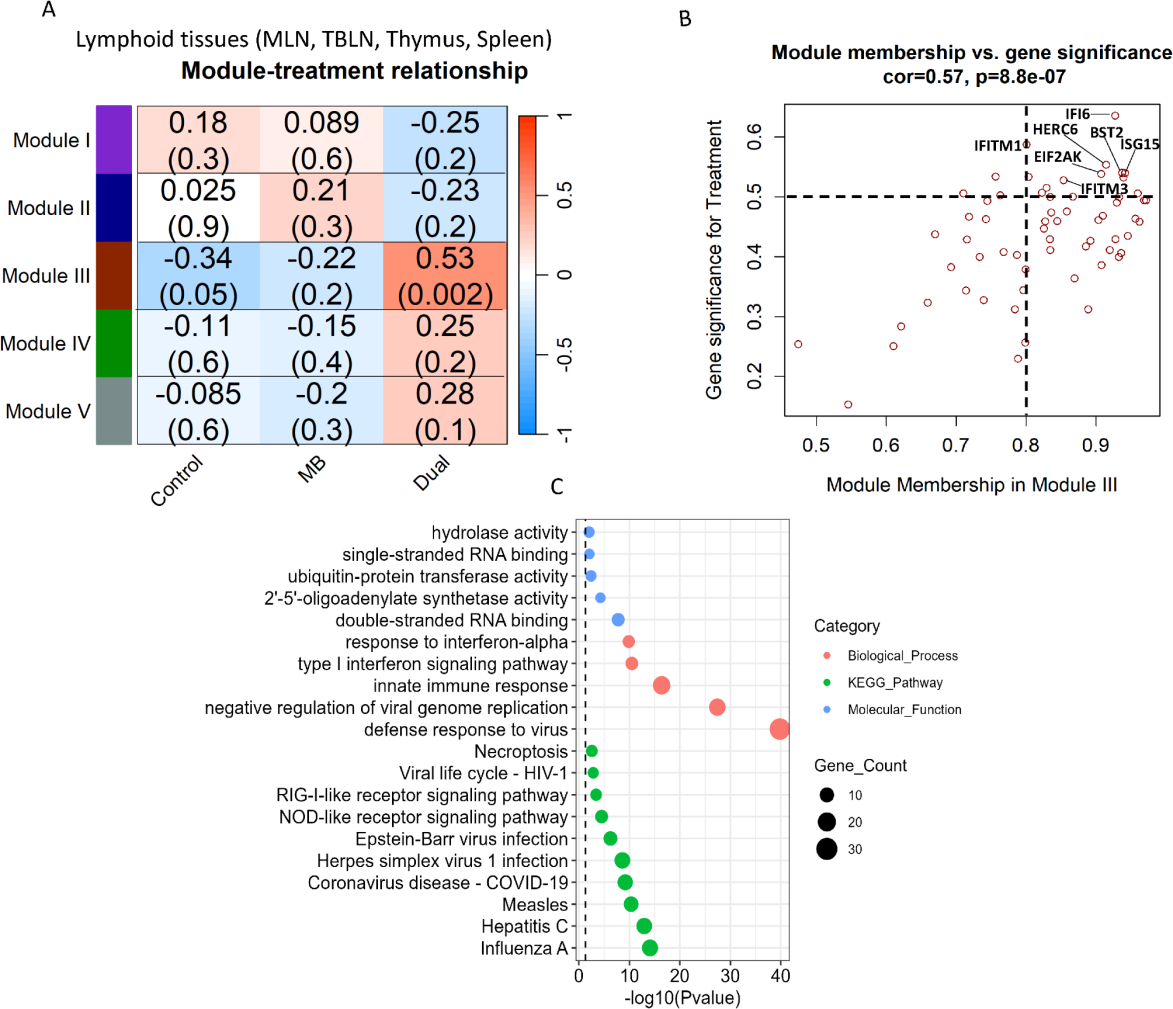
Weighted gene co-expression network analysis (WGCNA) in lymphoid tissues for Control, *M. bovis* (MB), and Dual groups. (**A**) Module-treatment relationship graph where each row represents the module eigen value and each column represents infection status. The cells within the matrix show the correlation coefficient and *p*-value. Modules were found in mesenteric lymph node (MLN), tracheal-bronchial lymph node (TBLN), thymus, and spleen. (**B**) Correlation between module membership (MM) and gene significance (GS) of Module III genes, where MM represents the correlation between gene expression and the module eigen values and GS represents the correlation between gene expression and co-infection status. (**C**) Biological processes, pathways, and molecular functions enriched with co-expressed genes in Module III. The y-axis indicates the gene ontology (GO) term and the x-axis indicates the -log10 *p*-value. The size of the dot indicates the number of co-expressed genes enriched in the GO term. Enriched GO terms with a *p*-value < 0.05 (dashed line) were considered significant. IFI6 = interferon alpha inducible protein 6; BST2 = bone marrow stromal cell antigen 2; HERC6 = HECT and RLD Domain Containing E3 Ubiquitin Protein Ligase Family Member 6; ISG15 = interferon-stimulated gene 15; IFITM1/3 = interferon-induced transmembrane proteins 1/3

Weighted gene co-expression network analysis (WGCNA) was applied to lymphatic tissues (MLN, TBLN, spleen and thymus) and grouped co-expressed genes into 5 Modules, in which only Module III showed a significant correlation (*r* = 0.53) (Fig. 7A). The 64 co-expressed genes in Module III were negatively correlated to Control and MB groups, but positively correlated to the Dual group. A significant positive correlation (*r* = 0.57) was also observed between the gene significance (GS) for treatment and module membership (MM) of Module III genes (Fig. 7B). The genes with the highest GS and MM in Module III included *IFI6*, HECT and RLD Domain Containing E3 Ubiquitin Protein Ligase Family Member 6 (*HERC6*), *LOC112441507* (ortholog of bone marrow stromal cell antigen 2/*BST2*), interferon-stimulated gene 15 (*ISG15*), and interferon-induced transmembrane proteins (*IFITM1* and *IFITM3*). *IFI6* had the highest association with infection status in Module III and was an upregulated DEG in thymus, spleen, TBLN, and liver of the Dual group. *ISG15* was also an upregulated DEG in spleen and MLN of the Dual group compared to Control or MB. *IFITM1* and *IFITM3* were upregulated DEGs in spleen and thymus of Dual compared to Control and MB. Co-expressed genes in Module III were enriched in pathways related to infection by other viruses (E.g. Influenza A, Hepatitis C, Measles, COVID-19) and biological processes associated with immune response and interferon signaling (Fig. 7C; Table 3).

**Table 3:** The most significant 10 pathways, biological processes, and molecular functions predicted to be correlated to infection status based upon co-expression in mesenteric lymph node (MLN), tracheal-bronchial lymph node (TBLN), spleen, and thymus

Category	Term	*P*-Value	Co-Expressed Genes
Pathway	Influenza A	8.2E-15	*OAS1Z*,* IRF9*,* IFIH1*,* PML*,* RSAD2*,* MX2*,* IFNG*,* DDX58*,* MX1*,* OAS1Y*,* OAS2*,* IRF7*,* EIF2AK2*,* STAT1*
	Hepatitis C	1.2E-13	*OAS1Z*,* LOC100139670*,* IRF9*,* RSAD2*,* MX2*,* IFNG*,* DDX58*,* MX1*,* OAS1Y*,* OAS2*,* IRF7*,* EIF2AK2*,* STAT1*
	Measles	4.4E-11	*OAS1Z*,* MX2*,* DDX58*,* OAS1Y*,* MX1*,* OAS2*,* IRF7*,* IRF9*,* EIF2AK2*,* IFIH1*,* STAT1*
	Coronavirus disease - COVID-19	6.6E-10	*OAS1Z*,* MX2*,* DDX58*,* OAS1Y*,* MX1*,* OAS2*,* ISG15*,* IRF9*,* C2*,* EIF2AK2*,* IFIH1*,* STAT1*
	Herpes simplex virus 1 infection	2.4E-09	*OAS1Z*,* LOC112441507*,* IRF9*,* IFIH1*,* PML*,* IFNG*,* DDX58*,* OAS1Y*,* LOC100298356*,* OAS2*,* IRF7*,* EIF2AK2*,* STAT1*
	Epstein-Barr virus infection	5.6E-07	*OAS1Z*,* DDX58*,* OAS1Y*,* OAS2*,* IRF7*,* ISG15*,* IRF9*,* EIF2AK2*,* STAT1*
	NOD-like receptor signaling pathway	3.2E-05	*OAS1Z*,* GBP1*,* OAS1Y*,* OAS2*,* IRF7*,* IRF9*,* STAT1*
	RIG-I-like receptor signaling pathway	3.9E-04	*DDX58*,* DHX58*,* IRF7*,* ISG15*,* IFIH1*
	Viral life cycle - HIV-1	1.4E-03	*MX2*,* LOC112441507*,* MX1*,* LOC100298356*
	Necroptosis	2.7E-03	*IFNG*,* ZBP1*,* IRF9*,* EIF2AK2*,* STAT1*
Biological Process	defense response to virus	1.5E-40	*LOC112444847*,* OAS1Z*,* IFIT2*,* LOC100139670*,* IFIH1*,* RSAD2*,* MX2*,* OAS1Y*,* MX1*,* LOC282255*,* LOC100298356*,* DHX58*,* ZBP1*,* ZNFX1*,* IFITM3*,* PRF1*,* LOC112441507*,* IFITM1*,* ISG20*,* IFIT5*,* IFNG*,* DDX58*,* IFI44L*,* OAS2*,* ISG15*,* IRF7*,* STAT1*,* IFI6*
	negative regulation of viral genome replication	3.8E-28	*IFITM3*,* OAS1Z*,* LOC112444847*,* IFITM1*,* LOC100139670*,* ISG20*,* RSAD2*,* IFIT5*,* MX1*,* OAS1Y*,* LOC282255*,* OAS2*,* ISG15*,* EIF2AK2*,* ZNFX1*
	innate immune response	4.1E-17	*LOC515676*,* TIFA*,* OAS1Z*,* LOC112441507*,* C2*,* HERC5*,* IFIH1*,* PML*,* RSAD2*,* MX2*,* DDX58*,* MX1*,* OAS1Y*,* LOC100298356*,* OAS2*,* DHX58*,* UBA7*,* IFI6*
	type I interferon signaling pathway	3.2E-11	*IFITM3*,* LOC112444847*,* OAS2*,* LOC282255*,* IRF7*,* IFITM1*,* STAT1*
	response to interferon-alpha	1.3E-10	*IFITM3*,* LOC112444847*,* MX2*,* LOC282255*,* IFITM1*,* EIF2AK2*
	response to interferon-beta	4.4E-08	*IFITM3*,* LOC112444847*,* LOC282255*,* IFITM1*,* XAF1*
	response to virus	7.9E-08	*RSAD2*,* MX2*,* MX1*,* OAS2*,* DHX58*,* IFIH1*
	positive regulation of interferon-beta production	1.2E-06	*OAS2*,* DHX58*,* IRF7*,* ISG15*,* IFIH1*
	negative regulation of viral entry into host cell	2.2E-05	*IFITM3*,* LOC112444847*,* LOC282255*,* IFITM1*
	interleukin-27-mediated signaling pathway	4.9E-05	*MX1*,* OAS2*,* STAT1*
Molecular Function	double-stranded RNA binding	1.7E-08	*OAS1Z*,* DDX58*,* OAS1Y*,* OAS2*,* DHX58*,* EIF2AK2*,* IFIH1*
	2’-5’-oligoadenylate synthetase activity	5.5E-05	*OAS1Z*,* OAS1Y*,* OAS2*
	ubiquitin-protein transferase activity	4.0E-03	*LOC509283*,* PML*,* LOC527520*,* HERC5*
	single-stranded RNA binding	8.4E-03	*DDX58*,* DHX58*,* IFIH1*
	hydrolase activity	9.5E-03	*DDX58*,* DHX58*,* IFIH1*,* TRANK1*
	RNA helicase activity	9.8E-03	*DDX58*,* DHX58*,* IFIH1*
	RNA binding	1.1E-02	*IFIT5*,* DDX58*,* DHX58*,* ZBP1*,* LOC100139670*,* IFIH1*,* ZNFX1*
	GTP binding	1.6E-02	*LOC783920*,* MX2*,* IFI44*,* GBP1*,* MX1*
	zinc ion binding	3.1E-02	*PML*,* ZCCHC2*,* DDX58*,* DHX58*,* IFIH1*,* ZNFX1*
	metalloendopeptidase inhibitor activity	4.2E-02	LOC112441507, LOC100298356

## Discussion

BRD is a highly prevalent disease in calves, in which mixtures of bacterial and viral pathogens are often isolated from lungs of affected animals. A primary viral infection can weaken the host immune system which then can lead to a secondary bacterial infection, resulting in BRD development. The present study uses transcriptome profiling of host response to *M. bovis* and BVDV co-infection to enhance our understanding of dynamic interactions between pathogens and their mechanistic effects in immune-related tissues.

Of the genes that were differentially expressed in liver of the Dual group compared to MB, several have established or alleged involvement in immune function. *ISM1* has been shown to promote expression of antiviral genes, such as interferons, and an increased expression of *ISM1* and interferon-stimulated genes (*IFI6* and *OAS1Y*) in Dual compared to MB in liver, suggests regulation of interferon signaling [27]. *IFI6* and *OAS1* are well-known interferon-stimulated genes that are strongly induced upon *interferon-α* treatment and this treatment in hepatic cell lines has been shown to reduce RNA levels of hepatitis B virus [28, 29]. Infection of mice with BVDV in a previous report demonstrated that BVDV antigen could not be detected in liver, which perhaps is related to clearance of the virus through interferon-stimulated genes [30]. BVDV infection appeared to influence interferon production and inflammatory mediators, such as chemokines, in a tissue specific manner, which could predispose the animal to bacterial infection. An upregulation of genes involved in amino acid metabolism in the liver of the Dual group compared to Control could indicate that the synthesis of certain amino acids supports production of immune-related proteins [31].

Increased expression of chemokine ligands (*CCL14*, *CCL16*, and *CXCL9*) and interferon stimulated genes (*OAS2*, *MX1*, and *ISG15*) in the TBLN of co-infected animals could demonstrate a proinflammatory response with potential activation of monocytes by *CCL16* and proliferation of leukocytes associated with increased *CCL14* levels [32, 33]. In addition, the increased expression of *IFI27* in TBLN of the Dual group could be associated with disease severity. Elevated expression of *IFI27* has been found in blood during respiratory syncytial virus and Influenza infection, and its upregulation in the respiratory tract of COVID-19 patients is associated with a higher viral load [34–36]. *IFI27* has also been shown to interact with RIG-I through RNA binding, which in turn impairs RIG-I activation and inhibits innate immune response [37]. Although *IFI27* could be an effective predictor of BRD severity, further research is needed to consider its association with BRD progression.

The upregulation of granzyme B in WBC of the Dual group compared to MB is an immune signature for lymphocyte activation, where granzyme B is mostly found in natural killer cells and cytotoxic T-cells. Previous work found that elevated levels of granzyme B results in increased blistering in autoimmune diseases and inhibition of granzyme B reduced blister fluids and lesions [38]. Perhaps upregulation of granzyme B contributes to inflammation and lesion development in the Dual group. In addition, the downregulation of *BOLA-DOB* in WBC of the Dual group may alter activation of antigen presentation and lead to immunodeficiency [39, 40]. Given that blood samples can be easily used for diagnostic tests, these DEGs may serve as putative biomarkers for infection severity in BRD.

Response to co-infection appeared to be tissue-specific in thymus and spleen due to their unique roles in the immune system. Tropism is described as the ability of a pathogen to infect a location or organ, where some pathogens are considered broadly tissue tropic because they infect most organs [41]. However, our results suggest that *M. bovis* and BVDV largely impact the transcriptome of the thymus and spleen compared to liver, WBC, and lymph node tissues (MLN and TBLN). In comparison to the control group, single infection with *M. bovis* had minor impacts on gene expression across tissues. It should be considered that *M. bovis* can lead to chronic infection and the present study focuses on gene dysregulation during early phases of infection. Previously, it was observed that Influenza D Virus inducts a quicker and stronger host response compared to *M. bovis*-infected calves [42]. Therefore, the extent of dysregulation found in thymus and spleen is likely an early indicator of immune system disruption due to viral infection and future work should include later time points to determine the effects of long-term *M. bovis* infection. Although tissue or organ infection status may change over the course of BRD, identifying targeted tissues at late stages of disease could aid in appropriate treatment.

The spleen plays a role in resistance and elimination of pathogenic microorganisms and ECM of the spleen provides a favorable environment for the development of an immune response [43]. In spleen, several ECM components (Type I, IV, and VI collagens, *LAMA4*, *HSPG2*, and *TNXB*) were downregulated in Dual compared to Control and MB. In the spleen of chicken, Newcastle disease virus and bursal disease virus both displayed EM degradation through reduced collagen levels, where the degradation of the EM was suggested to increase viral spread and subsequently viral load [44, 45]. Given that marginal zones of the spleen ECM function in antigen trapping and processing, perhaps damage to the ECM results in impairment of immune cell localization and contributes to immunosuppression [44, 46]. Additionally, the basement membrane of the ECM is a barrier to prevent invasion by microorganisms and downregulation of basement membrane maintenance proteins (*COL15A1*, *COL4A2*, and *HSPG2*) in the Dual group could allow successful host invasion in the spleen [47]. Genes involved in the PI3K/AKT signaling pathway were also downregulated in the Dual group in spleen and PI3K/AKT functions in regulating anti-apoptosis and autophagy. The PI3K/AKT pathway promotes cell survival and metabolism in response to extracellular signals, and inhibition of PI3K/AKT signaling has previously been linked to decreased expression of ECM components [48, 49]. BVDV can mediate the inhibition of AKT signaling, which leads to reduced proliferation of CD4 + T cells in cytopathic BVDV and induced apoptosis of CD4 + and CD8 + T cells in noncytopathic BVDV [50]. The depletion of CD4 + T cells through repressed AKT signaling in spleen may lead to increased viral load or prolong infection.

Co-infection in the thymus resulted in upregulation of cathepsins (*CTSB*, *CTSC*, *CTSS*, *CTSH*, *CTSZ*), which are mainly found in lysosomes and have been previously implicated in viral infection efficiency. For example, increased expression of cathepsin B (*CTSB*) has been suggested to support initial viral entry into target cells for Ebola virus glycoprotein-mediated infection and cathepsin S (*CTSS*) in SARS-CoV-2 entry [51, 52]. High levels of *CTSB* has also been associated with inhibition of major histocompatibility (MHC) class II antigen-processing pathways in Influenza A viral infection [53]. In addition to cathepsins, upregulation of *LAMP1* was observed in the Dual group and increased expression of *LAMP1* was found to enhance SARS-CoV-1/2 production in humans via enhanced exocytosis [54]. *LAMP1* has also been associated with lysosomal trafficking of classical swine fever virus (CSFV) during early stages of infection and upregulation of cathepsin C isoforms due to CSFV infection has also been observed [55, 56]. This may suggest that lysosome-associated genes, including cathepsins and *LAMP1*, could stimulate viral entry and production due to co-infection and may serve as effective targets for antiviral therapy.

Regulation of cell cycle progression was also significantly altered in thymus, in which cell cycle promoters (cyclin dependent kinases 1,2, and 6) were downregulated and a cell cycle inhibitor (*CDKN1A*) was upregulated in the Dual group compared to Control and MB. Inhibition of cell cycle progression has been observed in porcine respiratory II virus infection as well as coronaviruses, where viruses exploit host cell machinery to benefit their pathogenesis and arrest cells in a certain phase of the cell cycle to favor viral replication [57, 58]. Together, this suggests that BVDV reduces T-cell proliferation, which leads to thymic cortex reduction and T-cell depletion.

Activation of the complement system has been thought to influence the outcome of pestivirus infections. For example, the knockout of an activator of the complement system, *CD46*, has differing effects on cellular entry for pestiviruses, where knockout of *CD46* greatly reduces infection in *Pestivirus H* and causes minor reductions in infection in *Pestivirus G* [59]. Upregulation of complement component *C1Q* has also been found during infection with CSFV, where the increased expression of *C1Q* in spleen is suggested to enhance lymphocyte apoptosis and induce cytokine production [60]. However, upregulated *C1Q* was only found in the thymus of co-infected animals in the current study. The tissue-specific upregulation of *C1Q* may reflect differences in the pathogen’s tropism or immune strategies. *STAT1* was also upregulated in Dual compared to Control in thymus and evidence has suggested that *STAT1* positively regulates complement components in response to viral infection [61]. In the present data, co-infection triggered activation of complement and coagulation pathways. For example, *C3*, complement factor B (*CFB*), and complement 3a receptor 1 (*C3AR1*) were upregulated in the Dual group compared to Control and MB groups in thymus. *C3* activation causes upregulation of *C3AR1* and *CFB* stimulates the assembly of a *C3* convertase, which breaks *C3* to produce processed fragments, such as *C3a*. Previous work has shown hyperactivation of complement pathways in COVID-19, where high levels of *C3a* correlate with severity of COVID-19 infection [62–64]. Increased expression of *C3* and *CFB* in the Dual group would suggest the presence of a *C3* convertase to yield *C3a* fragments in thymus and the binding of *C3a* to *C3AR1* would drive inflammation [65]. Although COVID-19 and BVDV belong to different viral classes, hyperactivation of the complement pathway may play a parallel role in their pathogenicity [66]. Research has shown that complement components, such as *C5a*, can drive lymphocyte exhaustion following severe infections and induce apoptosis in thymocytes, and both COVID-19 and BVDV are associated with reduced thymic T-cell output and impaired thymic function [67]. Potentially, the use of complement inhibitory drugs would be therapeutically beneficial for BRD treatment.

Injury of endothelial cells is also associated with COVID-19 and elevated levels of endothelial cell injury markers (*VWF* and *THBD*) are often found in patients with severe COVID-19 [64, 68, 69]. In the current study, these markers were upregulated in thymus of the Dual group compared to Control and MB. Previous work has shown that high levels of *VWF* and *THBD* correlate with mortality in COVID-19 patients [70, 71]. MB and BVDV co-infection results in damage to thymic epithelial tissue that reduces thymic function and results in increased severity of disease through impairment of the blood-thymus-barrier.

WGCNA revealed co-expressed genes that were the most highly associated with infection status including: *IFI6*, *HERC6*, *ISG15*, *IFITM1*, and *IFITM3*. Villamayor et al. found that suppression of *IFI6* expression resulted in decreased Influenza A Virus and SARS-CoV-2 titers, which was suggested to be mediated by binding of *IFI6* with RIG-I that impacts RIG-I activation [72]. In the current study, *IFI6* was upregulated in thymus, spleen, TBLN, and liver of the Dual group, suggesting it may have a role as a negative regulator of innate immune response. As previously mentioned, *IFI27* is suggested to operate through a similar mechanism and upregulation of *IFI27* and *IFI6* may have a synergistic impact on RIG-I activation in TBLN. One study observed increased expression of genes related to interferon activity and viral defense, including *HERC6* and *ISG15*, in the blood of animals that died due to BRD [73]. *HERC6*, *IFI6*, and *ISG15* are often produced in host cells due to viral infection and have been suggested to be markers for animals needing BRD treatment [74]. In contrast, *IFITM1* and *IFITM3* have previously been shown to inhibit CSFV replication, which may suggest that the co-regulation of these genes in lymphoid tissues is done to combat BVDV and reduce its pathogenesis [75].

## Conclusion

The present data demonstrates tissue-specific host response to co-infection. Increased differential expression in immune related pathways within thymus and spleen suggests they are the primary dysregulated tissues in *M. bovis* and BVDV co-infection. Dysregulation of ECM-receptor interaction in spleen may be an immunosuppressive mechanism by the virus to escape host immune response and aberrant activation of complement pathways in thymus may lead to thymic atrophy. It is important to note that small sample sizes (*n* = 2–3) were used for the treatment groups in this study to focus on identifying robust alterations in gene expression. A larger number of biological replicates could offer insights into minor changes in gene expression resulting from the host response. The DEGs identified in this study could provide further insights into the mechanisms contributing to BRD mortality and aid in the development of biomarkers and therapeutic targets for treatment.

## Electronic supplementary material

Below is the link to the electronic supplementary material.

**Supplementary Material 1**: **Supplementary Table S1**: RNAseq processing. RNAseq processing statistics before trimming (raw reads), after trimming (trimmed reads) and mapped reads for each sample. **Supplementary Table S2**: RNAseq count matrix. RNAseq count matrix generated by featureCounts with gene ID, chromosome location, and gene length information for each sample. **Supplementary Table S3**: DESeq2 results. Results of differential expression analysis for Control vs. MB, Control vs. Dual, and MB vs. Dual in each tissue.

## Data Availability

Raw sequence files can be found under BioProject Accession number PRJNA1166195 (https://www.ncbi.nlm.nih.gov/sra/?term=PRJNA1166195).

## Declarations

### Authors’ information

R.A. and S.F. completed all work as National Animal Disease Center, USDA-ARS employees. The work of R.A. predates his employment at the Center for Veterinary Biologics, USDA-APHIS. The work of S.F. predates her employment at Auburn University College of Veterinary Medicine.
